# Disseminated *Mucor indicus* Infection in a Heart Transplant Recipient: Complementary Conventional and Sequencing-Based Diagnostic Approaches

**DOI:** 10.3390/jof12100767

**Published:** 2026-10-13

**Authors:** Konstantin Tanida, Nicole Degel-Broßmann, Alexis Robitaille, Denise Heim, Markus Glatzel, Patrick Blümke, Martin Aepfelbacher, Marc Lütgehetmann, Martin Christner, Adam Grundhoff, Nicole Fischer

**Affiliations:** 1 Institute of Medical Microbiology, Virology and Hygiene, University Medical Center Hamburg-Eppendorf, Martinistrasse 46, 20246 Hamburg, Germany; 2 Institute for Molecular Virology and Tumorvirology, University Medical Center Hamburg-Eppendorf, Martinistrasse 46, 20246 Hamburg, Germany; 3 Leibniz Institute of Virology, Martinistrasse 52, 20246 Hamburg, Germany; 4 Institute for Neuropathology, University Medical Center Hamburg-Eppendorf, Martinistrasse 46, 20246 Hamburg, Germany

**Keywords:** *Mucor indicus*, mucormycosis, heart transplantation, cerebral mucormycosis, metagenomics, next-generation sequencing, high-throughput sequencing

## Abstract

**Background:** Mucormycosis is a rapidly progressive and often fatal invasive fungal infection in immunocompromised patients. Diagnosis remains challenging because microscopy and culture have limited sensitivity, while targeted molecular assays may fail to detect uncommon *Mucorales* species. **Case Presentation:** We report a fatal case of disseminated *Mucor indicus* infection with pulmonary and cerebral involvement in a heart transplant recipient receiving immunosuppressive therapy and posaconazole prophylaxis. Initial microscopy of bronchial secretions demonstrated broad pauciseptate hyphae consistent with *Mucorales*, whereas targeted PCR assays remained negative. Species identification was subsequently achieved by culture, MALDI-TOF mass spectrometry, and internal transcribed spacer (ITS) sequencing. Following neurological deterioration, brain biopsy confirmed cerebral involvement histopathologically, and ITS sequencing detected *M. indicus*. Comparative DNA- and RNA-based metagenomic sequencing identified *Mucorales* sequences, although read abundance remained unexpectedly low despite extensive histopathological fungal burden. Nevertheless, independent mapping of metagenomic reads against databases comprising all genomic viral and fungal reference sequences readily identified *Mucor indicus* as the sole pathogen receiving significant scores. **Conclusions:** This case illustrates that targeted Mucorales PCR may fail to detect an uncommon species despite evidence of invasive fungal infection. Microscopy, culture, histopathology, ITS sequencing, and comprehensive metagenomic analyses provided complementary evidence for disseminated *Mucor indicus* infection. Integrated diagnostics combining histopathology, culture, and complementary molecular methods remain essential for accurate diagnosis of invasive mucormycosis.

## 1. Introduction

Disseminated mucormycosis is a life-threatening invasive fungal infection caused by filamentous fungi of the order *Mucorales*, primarily affecting immunocompromised individuals, including patients with hematologic malignancies, recipients of solid organ transplantation, and those with profound neutropenia or uncontrolled diabetes mellitus. The disease is characterized by angioinvasion, rapid tissue necrosis, and hematogenous dissemination, frequently involving multiple organs, most commonly the lungs and central nervous system (CNS) [1,2]. Despite advances in antifungal therapy and supportive care, disseminated mucormycosis remains associated with poor outcomes, with mortality rates exceeding 50% and approaching 90% in cases with CNS involvement or delayed diagnosis [3,4,5,6,7,8].

Timely diagnosis of mucormycosis remains challenging and typically relies on a combination of histopathology, culture, and molecular diagnostics [9]. Current guidelines from the European Confederation of Medical Mycology (ECMM) and the Mycoses Study Group Education and Research Consortium (MSGERC) recommend at least genus-level identification to inform antifungal management and species-level characterization to improve epidemiologic understanding and facilitate recognition of emerging pathogens [3]. However, molecular diagnosis based on targeted polymerase chain reaction (PCR) assays may be limited by primer coverage, as most assays are optimized for the most prevalent Mucorales species potentially failing to detect uncommon pathogens [10,11].

Next-generation sequencing (NGS), including shotgun metagenomic and targeted amplicon-based approaches, has emerged as a promising tool for unbiased fungal pathogen detection directly from clinical specimens [12]. Unlike targeted PCR, nucleic acid–based broad-range approaches such as pan fungal PCR [13] and NGS [14,15] can enable species-level identification even in diagnostically challenging settings, including low fungal burden, culture-negative samples, atypical clinical presentations, or infections caused by rare species not routinely included in diagnostic panels. Registry data from Europe indicate that while *Rhizopus* and *Lichtheimia* species account for the majority of mucormycosis cases, *Mucor* species, including *Mucor indicus*, represent a minority of infections and are reported predominantly in transplant recipients and patients with hematologic malignancies [3].

Here, we report a fatal case of disseminated *Mucor indicus* mucormycosis with pulmonary and cerebral involvement in a heart transplant recipient. This case highlights the diagnostic complexity of rare *Mucorales* infections, particularly in the setting of negative targeted PCR results, and illustrates the added value of sequencing-based approaches for identification in invasive fungal disease.

## 2. Material and Methods

### 2.1. Culture and MALDI-TOF MS for Fungus Identification

Fungal isolates were cultured on Sabouraud Glucose Chloramphenicol Agar (SAB-GC; Oxoid, Waltham, MA, USA) and incubated at 30 °C. Antifungal susceptibility testing was performed using gradient strips (bioMérieux, Marcy-l’Étoile, France) on RPMI agar, following the manufacturer’s instructions, with an inoculum equivalent to a 1.0 McFarland standard. MICs were read after 24 h of incubation at 35 ± 2 °C. Fungal identification was performed by matrix-assisted laser desorption/ionization time-of-flight mass spectrometry (MALDI-TOF MS) using the MALDI Biotyper™ smart system (Bruker Daltonik GmbH, Bremen, Germany). Samples were prepared using the mycelium transfer method according to the manufacturer’s recommendations. Spectra were interpreted using the MBT HT Filamentous Fungi IVD module and the MBT Filamentous Fungi library version 2022 (Bruker Daltonik GmbH).

### 2.2. Nucleic Acid Extraction

Human tissue samples as well as the Mucor indicus isolate were processed in 2 mL microtubes prefilled with ceramic beads (Precellys Lysing Kit; Bertin Technologies SAS, Montigny-le-Bretonneux, France) and 1 mL phosphate-buffered saline. Tissue homogenization was performed using the Precellys 24 Tissue Homogenizer (Bertin Technologies SAS). The respiratory specimen was pre-treated with Liquillizer (Seegene Germany GmbH, Düsseldorf, Germany) in a 1:1 dilution. For nucleic acid extraction, 200 µL lysates or pre-treated respiratory secretions or 1000 µL of CSF sample were transferred to the MagNA Pure 96 system and processed using the DNA and Viral NA Small Volume Kit (Roche Molecular Systems), following the manufacturer’s recommendations.

RNA concentration and integrity were assessed using the Agilent TapeStation system according to standard quality control procedures.

### 2.3. Mucorales Diagnostic PCR; ITS PCR

For *Mucorales*-specific diagnostic PCR, 5 µL of purified nucleic acid was added to 20 µL of a prepared master mix containing the QuantiFast Pathogen RT-PCR +IC Kit (Qiagen, Hilden, Germany), as well as target-specific forward and reverse primers and a minor groove binder (MGB) probe. The assay targeted the following *Mucorales* species: *Mucor* spp., *Rhizopus oryzae*, *Rhizopus microsporus*, *Rhizomucor pusillus*, *Lichtheimia corymbifera*, and *Lichtheimia ramosa* [16]. Reactions were performed in a 96-well plate format. Each run included one negative control (H_2_O) and one corresponding positive control for each target. PCR amplification was carried out on a LightCycler 480 II instrument (Roche).

The fungal internal transcribed spacer (ITS) region was amplified using the ITS4 and ITS5 primers described by White et al. [17], followed by Sanger sequencing.

### 2.4. Metatranscriptomic Library and DNA Library Preparation and Sequencing

Untargeted high-throughput short read sequencing was performed on both total RNA and DNA extracted from a brain biopsy. For RNA-Seq, total RNA was subjected to DNaseI treatment (Thermo Fisher Scientific, Waltham, MA, USA), and 10 ng of purified RNA was used for library preparation via the SMARTer Stranded Total RNA-Seq Kit v2—Pico Input Mammalian (Takara Bio Europe, Saint-Germain-en-Laye, France). For DNA-Seq, 25 µL of the total nucleic isolation (~200 ng DNA) was sheared with a Bioruptor (Diagenode, Seraing, Belgium) in 13 cycles of 30 s sonication followed by 30 s pauses. Subsequently, DNA libraries were generated from the fragmented DNA using the NEBNext Ultra II DNA Library Prep Kit (New England Biolabs, Ipswich, MA, USA) according to the manufacturer’s instructions. Libraries were sequenced on an Element Biosciences AVITI24 instrument. RNA libraries were sequenced as paired-end 2 × 75-bp reads, whereas DNA libraries were sequenced as paired-end 2 × 150-bp reads.

### 2.5. Metagenomic Short-Read Analysis

Samples were analyzed using an in-house pathogen detection pipeline combining mapping, k-mer, and de novo assembly approaches. Raw sequencing reads were first assessed for quality using FastQC (v0.12.1) prior to adapter and quality trimming with trim_galore (v0.6.10). Trimming removed short reads, low-quality bases, reads with excessive ambiguous nucleotides (N), and residual Illumina adapter sequences. The filtered reads were subsequently aligned to the human reference genome GRCh38 (ENSEMBL) using BWA-mem2 (v2.2.1) [18]. Only high-confidence alignments, defined as uniquely mapped reads with a mapping quality score (MAPQ) of 60, were retained using sambamba (v1.0.0) [19]. Reads that did not map to the human genome were extracted from the original FASTQ files using BBTools [20] and subsequently aligned to viral and fungal reference sequence databases using BWA-mem2. Viral and fungal reference sequences were downloaded from the viral and fungal release directories of the NCBI Reference Sequence (RefSeq) FTP repository on 16 March 2026. At the time of retrieval, the FTP repository corresponded to RefSeq release 233, dated 26 January 2026. The downloaded compressed sequence files were decompressed and then indexed using BWA-mem2 (v2.2.1) with the bwa-mem2 index command. NCBI Taxonomy identifiers were assigned to individual reference sequences by matching their accession.version identifiers against the nucl_gb.accession2taxid.gz and nucl_wgs.accession2taxid.gz mapping tables, which were downloaded from the NCBI Taxonomy FTP repository on the same date. Metadata regarding viral host range, genome type and putative pathogenicity were extracted from the International Committee on Taxonomy of Viruses (ICTV, https://ictv.global/, accessed on 8 October 2026) master species list and metadata resource, as well as the NCBI Virus (https://www.ncbi.nlm.nih.gov/labs/virus/vssi/#/, accessed on 8 October 2026) and Virus Host DB (https://www.genome.jp/virushostdb, accessed on 8 October 2026) databases (reference data 5 August 2026). Putative fungal pathogens were annotated according to the data provided by Thambugala and colleagues [21].

BAM files resulting from the alignment were processed to extract reference sequence id, reference length, read counts, and base coverage values at depth thresholds of 1, 10 and 100. Extracted values were subsequently aggregated on associated species-level taxonomy identifiers by summation of individual values. To focus on relevant hits, viral hits were filtered for viral genera or species infecting vertebrate species. For DNA-seq reads, RNA-viruses with no intermediate DNA state were furthermore excluded from downstream analysis, while DNA- and retrotranscribing viruses were included in both DNA-seq and RNA-seq analyses. All candidate species supported by at least 5 reads were retained for downstream analysis. A PMC (Poisson-distribution minimal coverage) score was calculated for each species hit by dividing the observed number of covered bases (*b_count*) by the minimal number of bases expected to be covered (*b_expect*) under a random read distribution model. *b_expect* values were calculated using the cumulative distribution function *ppois* from the stats package in R (v4.6.1) with the following parameters: *q* = 0, *lambda* = read_count*read_length/*ref_length*, *lower.tail* = FALSE, *log.p* = FALSE, where *read_count* is the number of reads mapping to a given species, *read_length* is the average *read_length* (150), and *ref_length* is the total length of the reference sequence(s). Robust outliers were detected by calculation of Median Absolute Deviation (MAD) of PMC values using the *mad* function from the R Stats package. Species surpassing a stringent threshold of 5× MAD from the median PMC were considered significant outliers. DNA-seq and RNA-seq mapping statistics, PMC scores, and MAD values for all fungal and viral species are provided in Supplementary Dataset S1.

In parallel to the read mapping approach described above, host-subtracted reads were subjected to k-mer-based taxonomic classification with Kraken2 (v2.1.3) [22], followed by abundance estimation using Bracken [23]. RNA-seq reads were additionally subjected to de novo assembly contig-based pathogen detection and subsequent BLAST searches using NCBI BLAST+ v2.10.0+ as previously described in DAMIAN [24] pipeline (note that we did not perform de novo assembly from DNA-seq reads because the substantially larger genomic search space reduces the depth and continuity required for reliable assembly).

## 3. Results

### 3.1. Clinical Presentation and Conventional Diagnostics

A man in his late 50 s underwent heart transplantation for ischemic cardiomyopathy and was started on tacrolimus, mycophenolate mofetil, and prednisolone, together with antimicrobial prophylaxis including cotrimoxazole and posaconazole, immediately after transplantation. Following an initially uncomplicated post-transplant course, acute cellular rejection prompted intensified immunosuppression with corticosteroid pulse therapy and antithymocyte globulin (ATG). Two weeks after transplantation, the patient developed fever and cough. Chest CT demonstrated pulmonary infiltrates without radiographic signs suggestive of invasive mould infection. Atypical pneumonia was suspected and empiric antimicrobial therapy with meropenem and vancomycin was initiated. Bronchial secretions obtained on day 1 demonstrated broad pauciseptate hyphae with rectangular branching on day 2 suggestive of *Mucorales* on direct microscopy. Targeted PCR assays for *Legionella* spp., *Chlamydia pneumoniae*, *Chlamydia psittaci*, *Mycoplasma pneumoniae*, *Aspergillus* spp., and a laboratory-developed *Mucorales* PCR panel remained negative.

**Figure 1:**
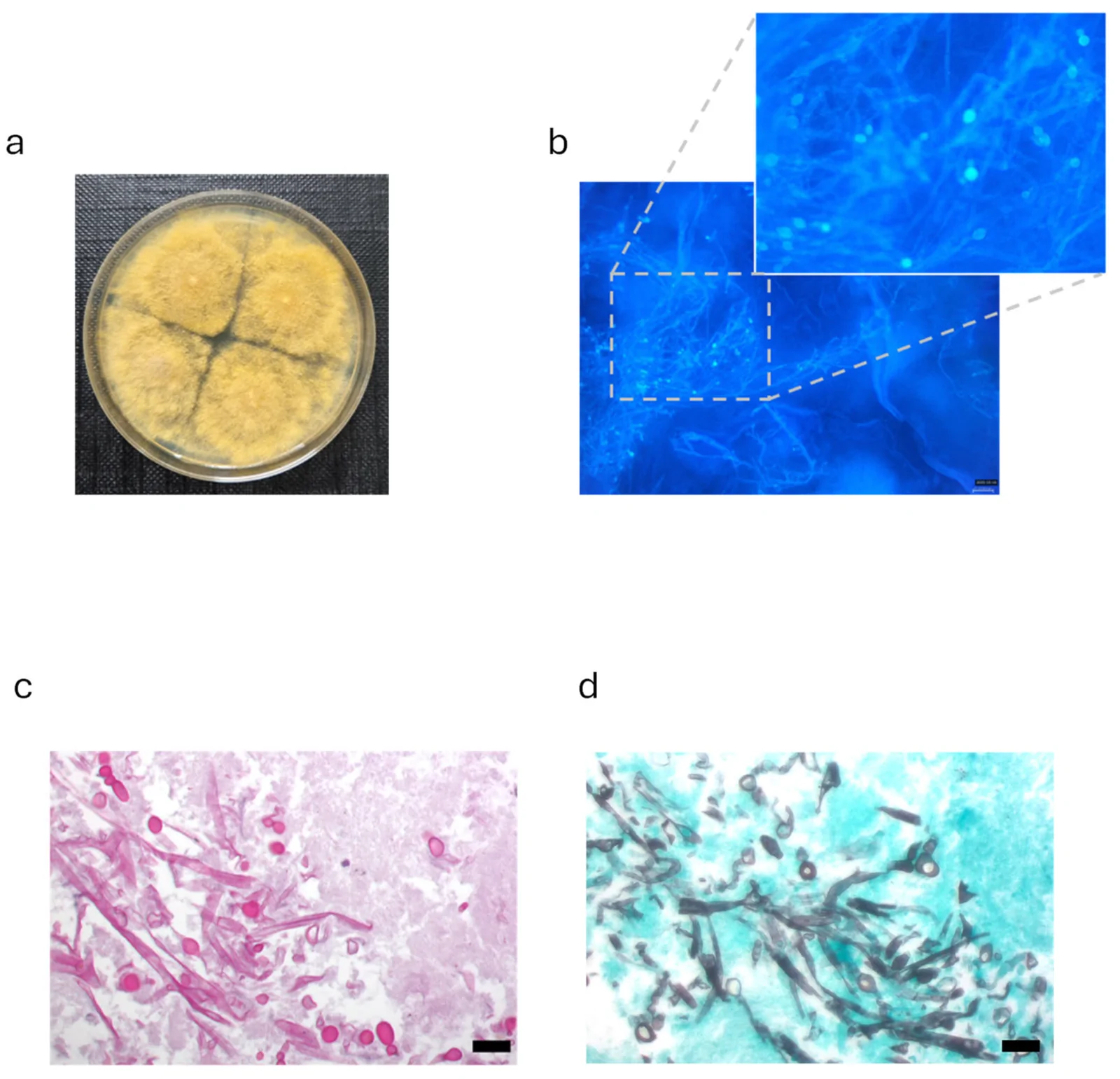
Microbiologic and histopathologic findings in disseminated *Mucor indicus* infection. (**a**) Macroscopic appearance of fungal colonies grown on Sabouraud agar at 30 °C. After incubation, colonies exhibited rapid growth with a cottony to floccose aerial mycelium and yellow pigmentation front and reverse. (**b**–**d**) Histopathologic examination of stereotactic brain biopsy tissue showing broad, ribbon-like, pauciseptate hyphae with occasional right-angle branching, consistent with *Mucorales*: (**b**) Calcofluor white staining (**c**), periodic acid–Schiff (PAS) stain and (**d**), Grocott methenamine silver (GMS) stain. Scale bar = 20 µm.

On day 2, fungal culture of bronchial secretions yielded rapid floccose growth (>10 colony-forming units) on Sabouraud agar (Figure 1a). *Mucor indicus* formed rapidly expanding colonies characterized by a woolly texture and deep yellow pigmentation on both the colony surface and reverse. Microscopic examination of the cultured isolate using lactophenol cotton blue staining showed morphological features consistent with *Mucor* spp. MALDI-TOF MS identified the isolate as *Mucor indicus* (score 1.78). This identification was subsequently confirmed by ITS sequencing, which demonstrated 99.8% sequence identity with the ITS region of *M. indicus* CBS 226.29 GenBank NR_077173 [25].

Antifungal susceptibility testing yielded MICs of 0.125 µg/mL for amphotericin B, 2 µg/mL for posaconazole, and >32 µg/mL for isavuconazole, as expected for this species [26]. Based on the initial microscopic detection of *Mucorales*-like hyphae, treatment with high-dose liposomal amphotericin B (10 mg/kg/day) was initiated immediately.

The patient suffered a stroke with severe right-sided hemiparesis and underwent thrombolysis, followed by cranial CT and MRI. Cranial MRI performed on day 3 demonstrated bilateral rounded lesions within the basal ganglia and additional corpus callosum involvement with homogeneous contrast enhancement. These findings were considered suspicious for an infectious etiology and, in the context of the established pulmonary *Mucor indicus* infection, raised concern for cerebral dissemination. Cerebrospinal fluid (CSF) analysis demonstrated elevated lactate and protein concentrations with granulocytic pleocytosis. Nevertheless, microbiological and molecular investigations, including broad-range 16S/18S rDNA PCR; pathogen-specific PCR assays for HSV-1, HSV-2, VZV, EBV, CMV, HHV-6, enterovirus, parechovirus, influenza A and B, JCV, measles virus, *Haemophilus influenzae*, *Listeria monocytogenes*, *Neisseria meningitidis*, *Streptococcus agalactiae*, and *Streptococcus pneumoniae*; and *Cryptococcus neoformans*- and Mucorales-targeted PCR, remained negative. In addition, fungal culture was performed and remained negative.

Cranial magnetic resonance imaging performed on day 3 demonstrated bilateral rounded lesions within the basal ganglia and additional corpus callosum involvement with homogeneous contrast enhancement, findings suspicious for hematogenous fungal dissemination.

Despite antifungal treatment, repeat bronchial cultures obtained on days 10 and 13 continued to yield *M. indicus* at low fungal burden (1 and 3 colony-forming units, respectively). On day 17, the patient developed a postoperative thoracotomy wound-healing disorder requiring vacuum-assisted closure therapy. Microbiological investigation of wound secretions and presternal tissue again yielded *M. indicus*, consistent with disseminated infection. Serum β-D-glucan assays remained negative on days 22 and 26.

Following neurological deterioration, stereotactic biopsy of bilateral basal ganglia lesions was performed on day 24. Direct microscopy using Calcofluor white staining demonstrated fungal hyphae (Figure 1b), and neuropathologic examination revealed periodic acid–Schiff (PAS)-positive and Grocott-positive hyphae-like structures consistent with invasive fungal infection (Figure 1c,d). Conventional fungal cultures of the biopsy specimen remained negative. Despite intensive antifungal therapy and repeated surgical wound debridement, the patient developed progressive disseminated infection and died on day 35. The clinical course and the results of the microbiological tests are chronologically outlined in Table 1.

### 3.2. Molecular and Metagenomic Analyses

ITS sequencing confirmed identification of the pulmonary isolate as *M. indicus*. In addition, ITS-PCR followed by sequencing detected *M. indicus* DNA in microscopy-positive CNS tissue despite negative fungal culture.

To further confirm the presence of *M. indicus* in brain-biopsy, we performed metagenomic sequencing of DNA and RNA. DNA-seq and RNA-seq generated 145,475,190 and 90,169,812 reads, respectively. After removal of human reads, the remaining reads were analyzed using three approaches: reference-based mapping with BWA-mem2, de novo assembly with DAMIAN [24], and k-mer-based classification with Kraken/Bracken [22,23].

For the first approach, we mapped our short-read data to fungal and viral sequences in the NCBI RefSeq database. In addition to reporting raw read counts for identified species, the pipeline calculates a PMC (Poisson-distribution Minimal Coverage) score that reflects the fraction of the reference that is covered, relative to an expected value from a random distribution model (see material and methods for details). The PMC score is especially useful for databases with heterogeneous length distribution such as the fungal RefSeq database, where unspecific reads mapping to long reference sequences may obscure true signals from short or incomplete sequence entries.

**Figure 2:**
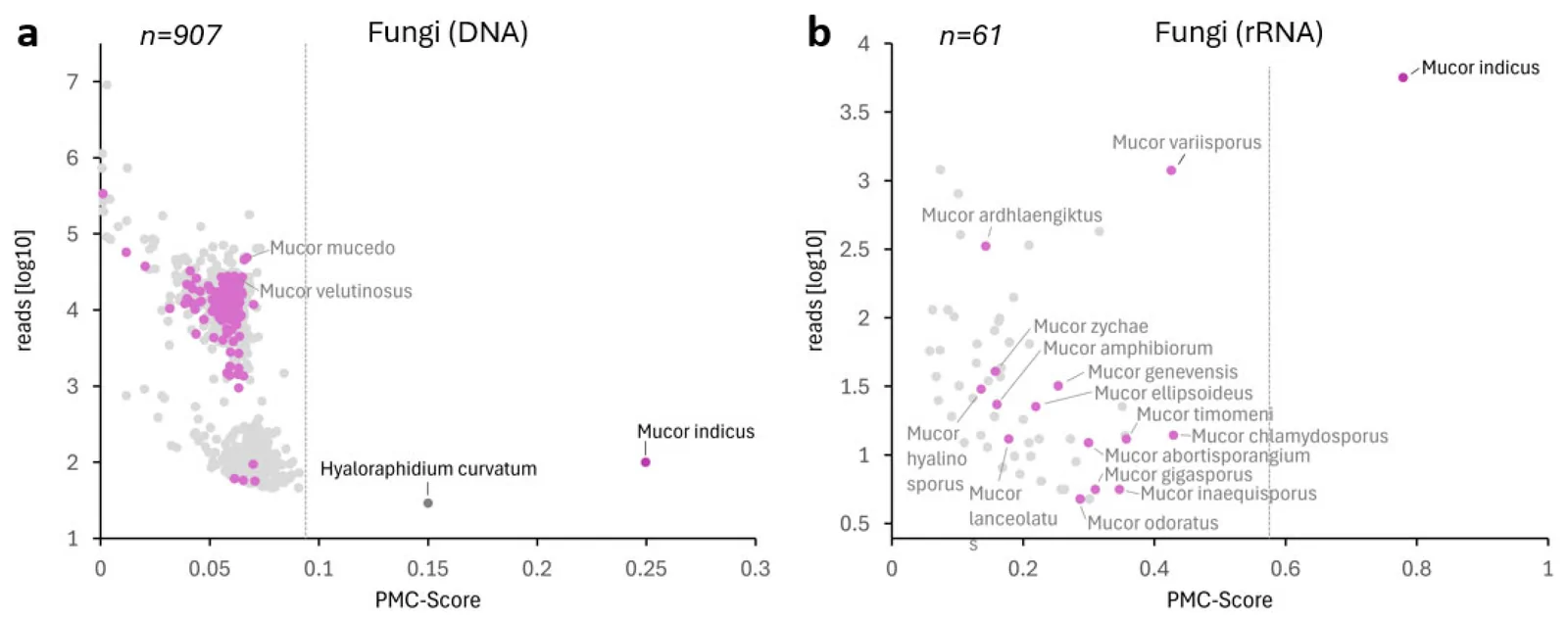
Results of metagenomic mapping to fungal RefSeq databases. Log-transformed read counts (vertical axis) and PMC score (horizontal axis) from metagenomic mapping of (**a**) DNA-seq reads to the fungal RefSeq database, (**b**) RNA-seq reads to ribosomal and ITS sequence of the fungal RefSeq database. The total number of detected candidate species supported by at least 5 reads is shown in the upper left of each panel. Species annotated as potential human pathogens (see material and methods for annotation sources) are shown in pink. The gray vertical dotted line in each plot represents the PMC outlier significance threshold (Median Absolute Deviation (MAD) threshold of 5; see material and methods for details). Outliers, as well as members of the family Mucoraceae are labeled with their species names.

In the DNA dataset, even though a total of only 100 reads mapped to its reference sequence, *M. indicus* was the highest scoring of only two outliers among fungal species (Figure 2a; Table 2). The second outlier, *Hyaloraphidium curvatum*, is a chytrid-like, free-living freshwater organism and therefore likely to represent a non-specific hit. For the RNA dataset, mapping was restricted to annotated fungal ribosomal RNA and ITS reference sequences to reduce the influence of heterogenous gene expression. *M. indicus* was the only significant PMC outlier and had the highest number of mapped reads (n = 5750; Figure 2b; Table 2). Reads assigned to other Mucoraceae species likely reflected cross-mapping to conserved ribosomal sequences.

Mapping of DNA and RNA reads to viral reference sequence databases only detected endogenous retroviruses and apathogenic Annellovirus species (see Supplementary Material), supporting the conclusion that *M. indicus* was the only pathogen responsible for the patient’s clinical symptoms. Complete fungal and viral mapping results are provided in Supplementary Dataset S1.

The reference-based mapping results were independently supported by DAMIAN de novo assembly, which recovered RNA-derived contigs assigned to *M. indicus* (Table 3). In contrast, Kraken/Bracken did not identify a significant Mucorales signal. Thus, ITS sequencing, reference-based mapping, and de novo assembly supported the presence of *M. indicus* in CNS tissue, whereas targeted Mucorales PCR and Kraken/Bracken classification remained negative.

## 4. Discussion

We report a fatal case of disseminated *Mucor indicus* infection with pulmonary and cerebral involvement in a heart transplant recipient receiving immunosuppressive therapy and antifungal prophylaxis with posaconazole. Human infection caused by *M. indicus* is rare and has been reported predominantly in immunocompromised hosts, with only isolated cases described in Europe [8,27,28]. This case highlights several clinically important challenges in mucormycosis, including rapid dissemination, diagnostic limitations of targeted molecular assays, and the complexity of managing rare *Mucorales* species in highly immunosuppressed patients.

Although mucormycosis is increasingly recognized among solid organ transplant recipients, most invasive infections are caused by *Rhizopus*, *Lichtheimia*, and *Rhizomucor* species, whereas *Mucor* spp. account for only a minority of cases [29]. The analytical coverage of targeted *Mucorales* assays depends on the primer/probe design and the sequence diversity represented during assay validation. Consequently, uncommon species may remain undetected even when they belong to a nominally covered genus. In our patient, repeated *Mucorales*-targeted PCR assays [16] remained negative in respiratory specimens despite microscopy demonstrating broad pauciseptate hyphae and subsequent culture-based identification of *M. indicus*, illustrating an important limitation of targeted diagnostics for rare fungal pathogens.

The clinical course was characterized by rapid progression from pulmonary infection to suspected central nervous system (CNS) involvement. Shortly after the initial pulmonary findings, magnetic resonance imaging demonstrated bilateral basal ganglia lesions and corpus callosum involvement, findings highly suggestive of hematogenous fungal dissemination. CNS mucormycosis is associated with particularly poor outcomes and remains diagnostically challenging. In our patient, CSF analyses showed marked inflammatory changes but repeatedly negative microbiologic and molecular testing, consistent with previous reports indicating limited sensitivity of CSF culture and PCR in CNS mucormycosis, particularly in the absence of leptomeningeal disease. Definitive evidence of cerebral involvement was ultimately obtained only through stereotactic biopsy, where fungal hyphae were identified histopathologically and *M. indicus* DNA was detected by ITS-PCR and subsequent sequencing. Cultures remained sterile, most likely due to the preceding, prolonged course of amphotericin B therapy.

An important finding of this case was the added diagnostic value of sequencing-based approaches, ITS-PCR sequencing but also next-generation sequencing (NGS), for confirming CNS involvement when conventional diagnostics remained inconclusive. While repeated targeted PCR assays failed to detect *M. indicus*, ITS sequencing of the pulmonary isolate enabled species identification, and agnostic metagenomic sequencing of CNS tissue consistently supported the presence of *Mucorales* DNA and RNA.

The performance of taxonomic classification pipelines varied, with Kraken/Bracken failing to identify significant K-mers mapping to *Mucorales*, whereas both reference-based mapping and de novo assembly readily identified *M. indicus* as the top-scoring fungal pathogen. While we do not know the exact reasons for negative fungal K-mer results, we suspect that the discordant performance of the different classification approaches may at least partly reflect differences in reference database composition, classification algorithms, and abundance thresholds. As already mentioned in the results section, despite having received high PMC scores in our mapping approach, the overall abundance of reads mapping to *M. indicus* was surprisingly low, especially considering the histopathological evidence of extensive fungal invasion. This is likely to be due to a combination of two effects: inefficient lysis of fungal cell walls during nucleic acid extraction, and incomplete *M. indicus* representation in the RefSeq database (the combined *M. indicus* reference length used in our analysis was approximately 64 kb, compared with a median fungal species reference length of approximately 26 Mb in the database; see Supplementary Dataset S1). Our observation underscores the importance of using metrics beyond absolute read abundance when evaluating diagnostic metagenomic data originating from complex samples. Such challenges may be particularly relevant in small brain-biopsy specimens, where the amount of tissue available for molecular analysis is inherently limited and nucleic-acid recovery may not directly reflect the fungal burden apparent histologically. The composite PMC score used here represents an efficient measure to eliminate false positives in such settings. Taken together, our metagenomic analysis results strongly support the diagnosis of *M. indicus* infection in CNS tissue, thus strengthening the biological plausibility of disseminated cerebral mucormycosis.

Another noteworthy aspect of this case was the occurrence of invasive mucormycosis during posaconazole prophylaxis [3,30,31]. The isolate had an amphotericin B MIC of 0.125 µg/mL, a posaconazole MIC of 2 µg/mL, and an isavuconazole MIC of >32 µg/mL. Neither EUCAST nor CLSI has established clinical breakpoints for Mucorales, so these MICs cannot be assigned formal susceptible or resistant categories. The very high isavuconazole MIC suggests limited in vitro activity, whereas the clinical significance of the posaconazole MIC of 2 µg/mL is uncertain. Posaconazole serum concentrations were not measured, as therapeutic drug monitoring is not routinely performed at our center. Consequently, adequate drug exposure during prophylaxis could not be confirmed, and insufficient exposure cannot be excluded as a contributing factor to breakthrough infection. Mucormycosis should therefore remain in the differential diagnosis even during mould-active prophylaxis.

Regarding treatment, Shen et al. reported a favorable outcome in a patient with disseminated *M. indicus* infection treated with liposomal amphotericin B combined with posaconazole [28]. However, this individual case cannot establish whether combination therapy would have changed the outcome in our patient.

Despite early initiation of liposomal amphotericin B and escalation to high-dose therapy, the patient developed progressive disseminated infection and ultimately died. The unfavorable outcome likely reflects the combination of profound immunosuppression, CNS dissemination, and the marked angioinvasive potential of mucormycosis. Early diagnosis and rapid initiation of antifungal therapy remain central to management; however, this case illustrates how diagnosis may be delayed when uncommon fungal species are not represented in targeted molecular assays.

## 5. Conclusions

This case highlights the diagnostic limitations of targeted molecular assays for uncommon Mucorales species. Negative targeted PCR results did not exclude invasive infection. Culture and ITS sequencing established the identity of the pulmonary isolate, while histopathology, ITS sequencing, and metagenomic analysis provided complementary evidence of cerebral involvement. These findings support combining conventional and sequencing-based fungal identification methods when clinical suspicion remains high despite negative targeted testing.

## Figures and Tables

**Table 1:** Temporal course of microbiologic and molecular diagnostics in disseminated *Mucor indicus* infection.

Day	Specimen	Diagnostic Findings	Clinical Interpretation
1	Bronchial secretion	PCR for *C. pneumoniae*, *C. psittaci*, *M. pneumoniae*, *Aspergillus* spp., and *Mucorales*: negative	No pathogen detected by PCR despite clinical suspicion
2	Bronchial secretion	Microscopy: broad, ribbon-like, pauciseptate hyphae; culture: >10 CFU *Mucor indicus*; MALDI-TOF MS: *M. indicus*	First microbiologic evidence of pulmonary mucormycosis
2	CSF	Elevated glucose, lactate, and protein; pleocytosis (58 leukocytes/µL, 92% granulocytes); PCRs (16S/18S rDNA, *Toxoplasma*, *Cryptococcus*, *Mucorales*): negative; fungal culture negative	Inflammatory CSF syndrome without molecular pathogen detection
3	Fungal isolate	ITS sequencing: 99.8% identity to *M. indicus*	Molecular confirmation of species identification
3	Antifungal susceptibility testing	E-test MICs: amphotericin B 0.125 µg/mL, posaconazole 2 µg/mL, isavuconazole > 32 µg/mL	Susceptibility pattern supportive of amphotericin B therapy
10	Bronchial secretion	Repeat culture: 1 CFU *M. indicus*	Persistent pulmonary detection despite therapy
13	Bronchial secretion	Repeat culture: low-count growth of *M. indicus* (3 CFU)	Ongoing respiratory persistence
17	Sternal wound secretion (VAC-treated site)	Low-count growth (1–3 CFU) of *M. indicus*	Evidence of postoperative soft tissue involvement
21	Presternal tissue	Growth of *M. indicus*	Persistent invasive infection
22; 26	Serum	β-D-glucan: negative	Expected negative biomarker result in mucormycosis
24	Brain biopsy	Calcofluor microscopy: hyphae; PAS/Grocott-positive fungal structures; culture: negative; ITS sequencing: *M. indicus*; NGS DNA sequencing: *M. indicus*; RNA sequencing *M. indicus*	CNS involvement confirmed despite culture negativity
26	Blood culture	1/2 bottles positive for *Escherichia coli*	Secondary bacteremia

**Table 2:** Significant hits resulting from mapping of DNA and RNA-seq reads against fungal RefSeq databases.

	DNA					RNA				
Fungal Species	Rank	Length	Covered	Reads	PMC	Rank	Length	Covered	Reads	PMC
*Mucor indicus*	1	63,741	3339	100	0.250	1	2341	1824	5750	0.779
*Hyaloraphidium curvatum*	2	34,680	614	29	0.150	-		-	-	-

Read mapping statistics of outliers as shown in Figure 2. Columns denote the following metrics: Rank: outlier rank according to PMC score; Length: Total reference sequence length (in bp); Covered: total number of bases covered at least once by mapped reads; Reads: number of mapped reads; PMC: Poisson-distribution minimal coverage score. See Supplementary Dataset S1 for comprehensive mapping information across all fungal species.

**Table 3:** Fungal species reported by the DAMIAN pipeline.

Contig			Blast Hit					
ID	Reads	Length	Accession	Title	Length	QCover	Perc. Ident.	E. val.
877393	33,871	2491	MH871967.1	Mucor indicus CBS423.71, 28S ribosomal RNA (partial)	1021	30%	99.12%	0.0
877587	4440	936	JNEK01000652.1	Mucor indicus B7402, 28S ribosomal RNA (partial)	535	57%	99.44%	0.0
878008	58	592	NC_084441.1	Mucor indicus mitochondrion, complete genome	61,400	100%	99.83%	0.0

De novo-assembled contigs from RNA-seq reads assigned to species hits by the DAMIAN pipeline. The ID and length of individual contigs, together with the total number of reads mapped back to each contig is shown in the left column. Right columns show positive BLAST alignment hits for each contig (QCover: percentage length of the contig sequence aligning with the subject, Perc. Ident.: percentage of identical aligned nucleotides; E. val.: e value for the alignment).

## Data Availability

The original sequencing read data presented in this study are openly available in the European Nucleotide Archive (ENA) under project accession number PRJEB127649. All fungal and viral mapping results are provided in Supplementary Dataset S1. Additional raw data cannot be made publicly available due to patient privacy and confidentiality requirements.

## Supplementary Materials

The following supporting information can be downloaded at: https://www.mdpi.com/article/10.3390/jof12100767/s1, Dataset S1: metagenomic read mapping results, Supplementary methods and results: viral metagenomic analysis; Supplementary Table S1: significant hits resulting from mapping of DNA and RNA-seq reads against viral RefSeq databases; Supplementary Table S2: Viral species reported by the DAMIAN pipeline; Supplementary Figure S1: results of metagenomic mapping to viral RefSeq databases.

## Abbreviations

Abbreviation	Full term
CSF	Cerebrospinal fluid
CT	Computed tomography
CFU	Colony-forming unit
CNS	Central nervous system
DAMIAN	Detection & Analysis of viral and Microbial Infectious Agents by NGS
ECMM	European Confederation of Medical Mycology
GMS	Grocott methenamine silver
HCCA	α-cyano-4-hydroxycinnamic acid
ITS	Internal transcribed spacer
MALDI-TOF MS	Matrix-assisted laser desorption/ionization time-of-flight mass spectrometry
MAPQ	Mapping quality
MBT	MALDI Biotyper
MIC	Minimum inhibitory concentration
MRI	Magnetic resonance imaging
MSGERC	Mycoses Study Group Education and Research Consortium
NCBI	National Center for Biotechnology Information
NGS	Next-generation sequencing
PAS	Periodic acid–Schiff
PE	Paired-end
RNA-Seq	RNA sequencing
VAC	Vacuum-assisted closure
VDB	Viral database
