# Supplementary material for: Disseminated Mucor indicus Infection in a Heart Transplant Recipient: Complementary Conventional and Sequencing-Based Diagnostic Approaches
Source: J Fungi (Basel). 2026 Oct 13;12(10):767. doi: 10.3390/jof12100767 (PMC13706590; doi:10.3390/jof12100767)
Supplement: Supplementary file 1 [file jof-12-00767-s001.zip › 261003_Mucor_indicus_supplementary Material.pdf]

Disseminated *Mucor indicus* infection in a heart transplant recipient: complementary conventional and sequencing-based diagnostic approaches

Tanida, K[1,#], Degel-Boßelmann, N[1,#], Robitaille A[2,3#], Heim, D[1], Glatzel, M[4], Blümke, P[3], Aepfelbacher M[1], Lütgehetmann, M[1], Christner, M[1], Grundhoff A[3*], Fischer N[2*]

Supplementary Materials:

Dataset S1 description

Supplementary methods and results

Supplementary Tables S1-S2

Supplementary Figure S1

Dataset S1: Metagenomic read mapping results

XLSX workbook with datasheets of species-level results from DNA-seq and RNA-seq read mapping to fungal and viral RefSeq databases. Tables columns are as follows: rank: rank according to PMC score; id: species name; ref_count: total number of RefSeq sequences assigned to species; tax_id: NCBI taxonomy id associated with species; root, realm/domain, kingdom, phylum, class, order, family, and genus: taxonomy lineage of the species; ref_length: the combined length of RefSeq sequences assigned to species; reads: total number of reads mapped to sequences assigned to species; bases_covered, bases_covered_10, bases_covered_100: total number of bases with minimal read coverage of 1, 10, and 100, respectively; fract_covered, fract_covered_10, fract_covered_100: the fraction of reference bases with minimal read coverage of 1, 10, and 100, respectively; pmc: poisson-distribution minimal coverage score; pmc_xmad: fold Median

Absolute Deviation (MAD) deviation from the median PMC score; outlier: boolean value indicating whether the species is considered a robust outlier (pmc_xmad >= 5).

Supplementary Methods and Results: Viral metagenomic analysis

Following removal of human reads, DNA-seq and RNA-seq reads from the brain-biopsy specimen were mapped to viral reference sequences from NCBI RefSeq using BWA-mem2. Viral reference sequences were downloaded on 16 March 2026, corresponding to RefSeq release 233. Viral host-range, genome-type, and putative-pathogenicity annotations were obtained from ICTV, NCBI Virus, and Virus–Host DB resources accessed on 5 August 2026.

Analysis was restricted to viruses infecting vertebrates. RNA viruses without a DNA intermediate were excluded from the DNA-seq analysis. Species supported by at least five reads were retained. Read counts, reference coverage, PMC scores, and outlier classification were calculated as described in the main Materials and Methods. RNA-derived contigs generated by the DAMIAN pipeline were additionally examined for viral sequence matches.

Reference-based mapping identified human endogenous retrovirus K and members of the Anelloviridae family in the DNA and RNA datasets (Supplementary Table S1; Supplementary Figure S1). DAMIAN likewise recovered RNA-derived contigs assigned to Anelloviridae (Supplementary Table S2). The detection of endogenous retroviruses as well as Anellovirus species (commensal viruses with near ubiquitous distribution) confirms the pipeline's ability to identify viral agents and supports the conclusion that no additional viral pathogen contributed to the patient's clinical representation.

Supplementary Table S1: Significant hits resulting from mapping of DNA and RNA-seq reads against viral RefSeq databases.

	DNA					RNA				
Viral Species	Rank	Length	Covered	Reads	PMC	Rank	Length	Covered	Reads	PMC
Human endog. retrovirus K	1	9,472	8,622	35,087	0.910	1	9,472	8,288	2,037	0.875
Alphatorquevirus homin19	2	3,808	3,415	678	0.897	2	3,808	1,635	109	0.435
Alphatorquevirus homin13	3	3,312	2,478	290	0.748	4	3,312	731	31	0.293
Alphatorquevirus homin18	4	3,313	2,336	315	0.705	-	-	-	-	-
Torque teno virus	-	-	-	-	-	3	10,184	682	17	0.302
Alphatorquevirus homin9	-	-	-	-	-	5	10,016	1,099	35	0.269
Alphatorquevirus homin15	-	-	-	-	-	6	7,605	1,101	63	0.204
Alphatorquevirus homin24	-	-	-	-	-	7	10,394	1,810	156	0.195
Alphatorquevirus homin5	-	-	-	-	-	8	3,229	503	38	0.188

Read mapping statistics of outliers as shown in Supplementary Figure S1. Columns denote the following metrics: *Rank*: outlier rank according to PMC score; *Length*: Total reference sequence length (in bp); *Covered*: total number of bases covered at least once by mapped reads; *Reads*: number of mapped reads; *PMC*: Poisson-distribution minimal coverage score. See Suppl. Dataset S1 for comprehensive mapping information across all viral species.

Supplementry Table S2: Viral species reported by the DAMIAN pipeline.

Contig			Blast Hit					
ID	Reads	Length	Accession	Title	Length	QCover	Perc. Ident.	E val.
616551	136	600	PV859134.1	Alphatorquevirus Alpha L5787_1346 gen. sequence	2,418	100%	89.60%	0.0
616370	44	701	MW455392.1	Anelloviridae sp. isolate P7-c1, complete genome	3,328	99%	97.70%	0.0

De novo-assembled contigs from RNA-seq reads assigned to viral species hits by the DAMIAN pipeline. The ID and length of individual contigs, together with the total number of reads mapped back to each contig is shown in the left column. Right columns show positive BLAST alignment hits for each contig (*QCover*: percentage length of the contig sequence aligning with the subject, *Perc. Ident.*: percentage of identical aligned nucleotides; *E. val.*: e value for the alignment).

Supplementary Figure S1: Results of metagenomic mapping to viral RefSeq databases.

(**a**) DNA-seq reads to a RefSeq database of vertebrate DNA and retrotranscribing viruses, and (**b**) RNA-seq reads to RefSeq vertebrate DNA, RNA and retrotranscribing viruses. The total number of detected candidate species supported by at least 5 reads is shown in the upper left of each panel. Species annotated as potential human pathogens (see material and methods for annotation sources) are shown in pink. The gray vertical dot ed line in each plot represents the PMC outlier significance threshold (Median Absolute Deviation (MAD) threshold of 5; see material and methods for details). Outliers, as well as members of the familiy *Mucoraceae* in (a) and (b), are labeled with their species names.
